# Prurigo Nodularis onset during secukinumab treatment of psoriasis: a case report

**DOI:** 10.1186/s13223-023-00811-5

**Published:** 2023-07-05

**Authors:** Qingqing Yang, Jiajie Lyu, Yu Gui, Shuling Yu, Jiajie Chen, Haoxue Zhang, Shengxiu Liu

**Affiliations:** 1 Department of Dermatology, the First Affiliated Hospital of Anhui Medical University, 218 Jixi Road, Shushan District, Anhui, Hefei, 230032 China; 2 Key Laboratory of Dermatology (Anhui Medical University), Ministry of Education, Anhui, 230032 Hefei China; 3 Inflammation and Immune-Mediated Diseases Laboratory of Anhui Province, Hefei, 230032 China

**Keywords:** Prurigo Nodularis, Secukinumab, Psoriasis, Adverse Reaction

## Abstract

**Background:**

Secukinumab has been approved by the U.S. FDA and the European Medicines Agency for the treatment of moderate-to-severe plaque psoriasis and psoriatic arthritis with the documented adverse effects. Here we reported in one case that a new symptom, Prurigo Nodularis (PN), developed during the programmed dosing of secukinumab.

**Case introduction:**

A 22-years-old male with a 6-month history of severe plaque psoriasis vulgaris was presented to the dermatology clinic two weeks after the fifth serial weekly doses of secukinumab, for the reason of the outbreaks of multiple erythematous papules and pruritus nodules on the trunk and extremities. Physical examination showed that psoriatic rash were under effective control with the previous targeted therapy of secukinumab for plaque psoriasis vulgaris, but new dermatologic condition was spotted with multiple edematous red firm papules on the trunk and extremities, in the form of soy or hemispherical nodules, red in color, firm to touch, with some ulcerated crusts visible at tops, but negative Auspitz sign. Pathological examination confirmed these papules as PN.

**Conclusion:**

This case report is shared to inform clinicians about an unannounced adverse effect of the secukinumab in the treatment of psoriasis, and it is recommended that patients be carefully informed of the possible risk of PN before starting treatment.

## Introduction

Secukinumab is a full-human monoclonal antibody and a targeted inhibitor of IL-17 A, a key factor in the Th17/IL-23 inflammatory pathway in psoriasis, and is approved for the treatment of moderate-to-severe plaque psoriasis. Here we represent a one case report that the occurrence and recession of PN were specifically related to the consecutive administration and withdrawal of secukinumab during the treatment procedure of psoriasis, indicating that secukinumab may induce PN, at least in Chinese individuals. The secukinumab induced PN was manageable, in which PN caused lesions and itching in the patient showed gradual regression and disappearance when the patient was discontinued the secukinumab administration and applied to a combined treatment of the antihistamine and 0.1% triamcinolone acetonide cream.

## Case report

**Fig 1:**
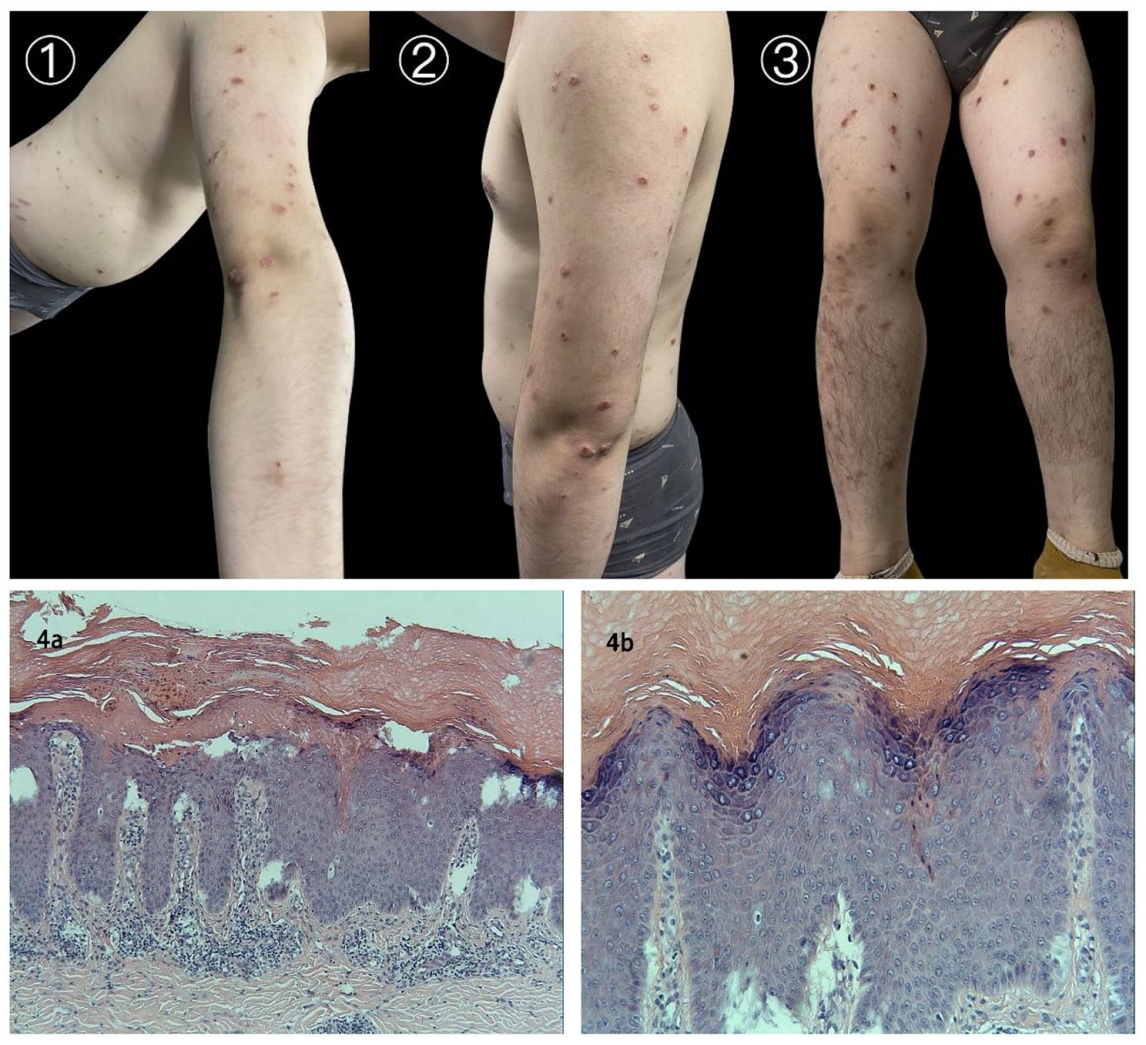
Figures 1, 2 and 3 show the multiple edematous red solid papules on the extensor side of extremities, in the form of soy or hemispherical nodules, with some visible as ulcerated crusts, firm to touch. Figure 4a (HE staining, x10), Fig. 4b (HE staining, x40) show the typical pathological features of PN in patient’s biopsy, with dense epidermis, hyperkeratosis and acanthosis and vascular proliferation and dilatation in the dermal papilla and the reticular layer as well as mild infiltration of peritubular inflammatory cells, mainly lymphocytes, histiocytes, and plasma cells

Patient is a 22-years-old male with a 6-month history of psoriasis, signed with the typical vulgaris on the head, neck, trunk, and extremities with a Psoriatic Area and Severity Index (PASI) of 26.1 and Body Surface Area (BSA) that involvement of 50%. First round psoriasis therapy using tacalcitol ointment/10 g, 0.02% clobetasol 17-propionate cream twice daily as well as cyclosporine/20 mg/day orally for 4 months showed no improvement on rash recoveries in the patient. The patient was then treated with secukinumab at a programmed schedule, 300 mg once a week at weeks 0, 1, 2, 3, and 4, followed by another 300 mg/ every 4 weeks. After the fifth Secukinumab injection at week 4, the patient experience significant improvement of the psoriatic lesion, with the PASI score decreasing by more than 90%. The patient was presented to the dermatology clinic 2 months after the starting secukinumab for evaluation of multiple erythematous papule and nodules that had erupted on his trunk and extremities. These lesions were described as intensely pruritic. The patient previous history had no record for similar symptoms. Patient also denied mosquitos and bedbug bites or a history of infectious diseases. Physical examination observed multiple edematous red solid papules on the trunk and extremities, in the form of soy or hemispherical nodules, with some tops visible as ulcerated crusts, red in color, firm to touch, and negative for Auspitz sign (Figs. 1, 2 and 3).

Laboratory tests in our hospital showed that no increase in serum leukocytes and eosinophils and that the indexes for functions of liver and kidney, as well as tuberculosis antibodies were normal. To exclude the allergenic pathogenesis, Serum IgE level tested was 498 U/mL, and allergens text 20 (shown in the annex) were tested all negative with a method of immunoblotting (Haooubo Company). The histopathology revealed (Fig. 4a and b) a dense epidermis, hyperkeratosis, and acanthosis. Vascular proliferation and dilatation were also seen in the dermal papilla and the reticular layer, accompanied with mild infiltration of peritubular inflammatory cells, mainly lymphocytes, histiocytes, and plasma cells. The lesions were clinically consistent with a diagnosis of PN, therefore, we prescribed to a treatment with ebastine 10 mg/day, desloratadine tablets 5 mg/day orally, and 0.1% triamcinolone acetonide cream twice a day for 3 weeks. After one month, the nodular lesions and pruritus gradually improved in the patient with the above treatment procedures. Following clinical improvement, the patient was informed about the risk of PN exacerbation with repeated uses of secukinumab, but he still insisted to acquire the sixth secukinumab injections for cure of psoriasis. He was dosed the sixth secukinumab for 300 mg at 4 weeks after the developing of PN. After this injection, the PN returned, and remained severe up to his seventh secukinumab injection—his secukinumab was discontinued at this point. And then a combined treatment was again applied, of which demonstrated noticeable therapeutic results in previous treatment, with ebastine 10 mg/day and levocetirizine dihydrochloride tablets 5 mg/day orally, 0.1% tacrolimus ointment twice a day, 0.1% triamcinolone acetonide cream, used twice a day. Follow up at 6 months after his last secukinumab injection, the patient no longer had any lesions consistent with PN, nor any psoriatic lesions.

## Discussion

Psoriasis is a common and easily recurring chronic inflammatory skin disease, with lesions mostly involving the skin, nails and joints, and is more prevalent in young adults. Environmental, immunological and genetic factors have been implicated in etiology of psoriasis. Although the exact ethology of psoriasis is unclear, IL-17 A is considered to play a significant role in the pathogenesis of patients with psoriasis [1]. Secukinumab, a full-human monoclonal antibody that is expressed in the Chinese hamster ovary cell line, belongs to the IgG1/κ isotype subclass, selectively binds to IL-17 A with high affinity, and neutralizes the biological activity of this cytokine [2]. Several randomized controlled trials have demonstrated the thrilling efficacy and safety of secukinumab in moderate-to-severe plaque psoriasis, with the most common adverse effects being nasopharyngitis, headache, upper respiratory tract infection, pruritus, diarrhea, neutropenia, hypertension, and arthralgia [3–6].

PN is a skin disease characterized by papules, nodules, and itching. The characteristic lesions appear as verrucous nodules and papules, and are typically distributed to the extensor surfaces of the lower extremities. The ethology of PN remains unknown, but current research suggests that its pathogenesis is related to neurological factors (increased expression of substance P and calcitonin gene-related peptide), immune factors (include Th2, Th1, Th-22, IL-17, IL-31), infectious factors, psychosomatic factors and tumor factors [7–9]. In this case, the patient developed papules and nodular lesions with severe itching during the use of secukinumab, which worsened during the recontinued use of secukinumab. Notably, the rash improved after discontinuing secukinumab, topical steroid creams and antihistamines. Skin testing done to common aeroallergens were all negative, to ensure no concomitant allergic rhino-conjunctivitis. Based on all the clinical characteristics, laboratory tests, histopathology, and history of medications, a diagnosis of drug related PN was confirmed in this patient. Adverse reactions such as atopic dermatitis (AD) [10], eczematous drug eruption [11], and perianal dermatomycosis [12] have been reported in the previous literature during the treatment of psoriasis with secukinumab. However, no case has been reported in PN during secukinumab treatment.

It is well known that psoriasis is a Th1/Th17 axis-mediated inflammatory responses, and eczema and AD are Th2 axis-mediated inflammatory responses. Although there is currently no evidence of a clear association between autoimmune disease and PN, it is reported that PN may be associated with Th1 and Th2. Fukushi et al[13] used relevant immunostaining methods to detect the cytokine signatures in 22 cases of PN, and the result revealed that 19 cases had anti-pSTAT6 antibody coloration in the whole epidermal nucleus, 21 cases demonstrated nuclear staining with anti-pSTAT3 antibody, pSTAT1 was expressed in the epidermal nuclei of 8 cases, 6 cases were positive for HLA-DR membrane expression, HABP staining was present in 21 cases, and pSTAT6 and pSTAT3 were expressed in the epidermal KC nucleus of most PN lesions. These data suggested that Th2 cytokines associated with STAT6 activation together with some unknown stimuli that activate STAT3 play a major role in the pathogenesis of PN. The expression of pSTAT1 in some lesions indicated that Th1 cytokines are involved in some aspect of pathogenesis of PN. Micah Bel et al [8] demonstrated that PN have a markedly cyclic and cutaneous Th22/IL-22 immune dysregulation and Th22 upregulation correlated with pruritus severity. Research data showed that IL-17 expression in patients with PN is decreased and suggested that increased Th17 signaling in the skin is primarily a local catalyst for Th22-mediated inflammatory responses. The Th1 and Th2 pathways are closely related in the immune response, and when the Th1 response is blocked or diminished, the Th2 response is conversely increased, as an antisense [14]. For example, one patient with psoriasis vulgaris developed AD lesions 4 days after each injection of secukinumab [10] and in a prospective study of 289 psoriasis patients treated with ixekizumab and secukinumab demonstrated eczematous drug eruption in 2.8% of patients [11]. The cause was suggested to be the imbalance of Th2/Th22 response to anti-IL-17 A drugs by blocking the Th1/Th17 pathway. Filomena Russo et al [15] also report a case of psoriasis during treatment of PN with dupilumab (IL-4 inhibitor) after 4 weeks, but authors claim that the pathophysiology of psoriasis induced after treatment with dupilumab is unclear. Remarkably, IL-4 is a negative regulator of Th1 and Th17 cells, which can inhibit the secretion of IL-1b and IL-6 by psoriasis epidermal cells. Therefore, the authors believe that blocking the Th2 response by targeting the IL-4 signaling pathway may lead to Th1/Th17 phenotypic transition, resulting in an inflammatory cytokine cascade, ultimately showing psoriasis skin lesions. In this case, the patient developed PN after treatment with a secukinumab for 2 months, the author speculated that the etiology was mainly due to secukinumab blocking the Th1/Th17 pathway, thereby causing an imbalance in the Th2/Th22 response, and thus inducing the development of PN. In addition to the consideration of drug factors leading to immune imbalance and thus triggering the development of PN, it is also necessary to rule out an allergy to the secukinumab drug itself. The clinical manifestations of drug rash are diverse, with rapid development and short incubation period. In addition of pruritus, systemic symptoms include fever, headache, diarrhea, increased leukocytes and eosinophils in the serum, and a positive drug-induced lymphocyte stimulation test (DSLT). The patient had a nodular papular rash on the extremities with a slow onset, no fever or organ damage, and negative serum and tissue eosinophils. Unfortunately, we were unable to perform a DSLT to confirm that it is not drug allergy reaction, but clinical manifestations and histopathology together enabled to clarify the diagnosis of PN.

The development of PN during the treatment of psoriasis with the secukinumab is clinically rare, and the etiology may be related to a Th22/IL-22 immune response imbalance, and the clear pathogenesis needs to be further investigated. However, only one case of PN was found in our outpatient clinic, so the exact pathogenesis needs to be further investigated. This case is shared to inform clinicians about yet another adverse effect of the secukinumab in the treatment of psoriasis and to suggest that patients should be asked about their history of AD, PN, and eczema before starting biologic agents, and even if they do not have a history of these conditions, it is recommended that they be informed as carefully as possible about the potential risks involved.

## Data Availability

Not applicable.

## Abbreviations

PN: Prurigo Nodularis
FDA: Food and Drug Administration
IL-17 A: Interleukin-17 A
PASI: Psoriatic Area and Severity Index
BSA: Body Surface Area
AD: Atopic Dermatitis
DLST: Drug-induced Lymphocyte Stimulation Test
IL-22/IL-31/IL-4: Interleukin-22/ Interleukin-31/ Interleukin-4
Th22/Th17/Th2/Th1: T helper 22 cell/ T helper 2 cell/ T helper 1 cell/ T helper 17 cell
pSTAT6/ pSTAT3/ pSTAT1: Phosphorylated signal transducer and activator of transcription 6/3/1
HABP: Hyaluronic acid binding protein
HLA: Human leukocyte antigen
KC: Keratinocyte
